# Clinical impact of estradiol/testosterone ratio in patients with acute ischemic stroke

**DOI:** 10.1186/s12883-021-02116-9

**Published:** 2021-02-26

**Authors:** Jung-Won Choi, In Woo Ryoo, Jun Yeong Hong, Kyung-Yul Lee, Hyo Suk Nam, Won Chan Kim, Seung-Hun Oh, Jaeku Kang, Hoi Young Lee, Sang-Jun Na, Ji Hoe Heo, Kee Ook Lee

**Affiliations:** 1 Department of Neurology, CHA Bundang Medical Center, CHA University, 59, Yatap-ro, Bundang-gu, Gyunggi-do 13496 Seongnam, Republic of Korea; 2 Department of Neurology, Konyang University College of Medicine, Daejeon, Korea; 3 Department of Neurology, Yonsei University College of Medicine, Seoul, Korea; 4 Myunggok Medical Research Institute, Konyang University College of Medicine, Daejeon, Korea

**Keywords:** Sex hormones, estradiol/testosterone ratio, Acute ischemic stroke, Functional outcome, Men

## Abstract

**Background:**

Sex hormones may be associated with a higher incidence of ischemic stroke or stroke-related events. In observational studies, lower testosterone concentrations are associated with infirmity, vascular disease, and adverse cardiovascular risk factors. Currently, female sexual hormones are considered neuroprotective agents. The purpose of this study was to assess the role of sex hormones and the ratio of estradiol/testosterone (E/T) in patients with acute ischemic stroke (AIS).

**Methods:**

Between January 2011 and December 2016, 146 male patients with AIS and 152 age- and sex-matched control subjects were included in this study. Sex hormones, including estradiol, progesterone, and testosterone, were evaluated in the AIS patient and control groups. We analyzed the clinical and physiological levels of sex hormones and hormone ratios in these patients.

**Results:**

The E/T ratio was significantly elevated among patients in the stroke group compared to those in the control group (*P* = 0.001). Categorization of data into tertiles revealed that patients with the highest E/T ratio were more likely to have AIS [odds ratio (OR) 3.084; 95% Confidence interval (CI): 1.616-5.886; *P* < 0.001) compared with those in the first tertile. The E/T ratio was also an independent unfavorable outcome predictor with an adjusted OR of 1.167 (95% CI: 1.053-1.294; *P* = 0.003).

**Conclusions:**

These findings support the hypothesis that increased estradiol and reduced testosterone levels are associated with AIS in men.

## Background

Sex hormones act on the brain via both genomic and non-genomic receptors [1]. Previous preclinical evidence had shown that sex hormones play an important neuroprotective role following acute ischemic stroke (AIS) and other acute central nervous system conditions such as spinal cord and traumatic brain injuries [2]. Sex hormones exert their effects directly on neuronal death pathways, and indirectly by modulating the immune system. The effects of these hormones on stroke outcomes are dose-dependent and are further associated with the patient’s age [3]. Age has been linked to the prediction of cardio-metabolic risk. Sex hormones play multiple roles in the development of cardiovascular disease [4]. There are similarities and differences in sex hormones between males and females. Aging has a significant impact on estrogen and androgen levels in both sexes. In particular, while estrogen levels decrease drastically only in post-menopausal women, androgen levels gradually begin to decrease around 30 years of age in both sexes [5–7].

Interestingly, both clinical and experimental evidence suggest that androgen levels drop after ischemic stroke. This raises the question of whether ischemia-induced reduction in androgen levels may be as important as the steady levels of hormones prior to the insult. Some recent bench studies have revealed conflicting data and indicate that androgens can either prevent or exacerbate ischemic damage [8].

The classical endogenous and exogenous sex hormones (estrogen, testosterone, and progesterone) have profound effects on stroke incidence, risk factors, and various outcomes. Few studies have examined the direct or indirect effects of sex hormones in the treatment of ischemic stroke [9–11]. Although the available evidence supports the hypothesis that sex hormones may be associated with the pathophysiology of AIS, it remains to be determined whether these hormones affect the acute stage of the condition. The goal of this study was to (1) determine the physiological levels of the principal sex hormones in AIS; (2) investigate the relationship between the possible sex hormone ratio including estradiol/testosterone (E/T) ratio, estradiol/free testosterone (E/free T) ratio, and AIS; and (3) investigate the association of sex hormones and these ratios with functional outcomes after stroke.

## Methods

### Study population

The AIS patient group consisted of 146 consecutively enrolled male patients who had been admitted within 48 hours of the onset of symptoms to our neurology department from January 2011 to December 2016. The control group consisted of 152 age-matched neurologically healthy males without any recent history of stroke or other thrombotic events. The control participants were recruited from the outpatient clinic of our hospital during their scheduled health examinations. This study was conducted according to the guidelines of the Declaration of Helsinki and approved by the institutional human research ethical committee (IRB approval no. 2011-004). Informed consent was obtained from all participants.

### Risk factor assessment

We assessed the vascular risk factors including atrial fibrillation, cardiovascular disease (such as coronary heart disease, heart failure, and peripheral arterial disease), hypertension (HTN), diabetes mellitus (DM), hyperlipidemia, chronic kidney disease (CKD), and current smoking and alcohol drinking habits, in all participants. HTN was diagnosed when a patient demonstrated systolic blood pressure (SBP) ≥ 140 mmHg, diastolic blood pressure (DBP) ≥ 90 mmHg, or was receiving antihypertensive treatment. Diabetes was diagnosed when a patient had fasting plasma glucose level ≥ 7.0 mmol/L, hemoglobin A1c (HbA1c) ≥ 6.5 %, or had been receiving oral hypoglycemic agents or insulin [12]. We also determined serum levels of total cholesterol, high-density lipoprotein (HDL) cholesterol, low-density lipoprotein (LDL) cholesterol, triglycerides (TG), and estimated glomerular filtration rate (eGFR). Body mass index (BMI) was determined as the measured weight in kilograms divided by the measured height in meters squared. Hyperlipidemia was diagnosed as total cholesterol level > 6.21 mmol/L, LDL cholesterol > 4.14 mmol/L, or in patients receiving lipid-lowering medication. We did not include patients with severe hepatic dysfunctions (alanine aminotransferase or aspartate aminotransferase levels > 2 times the upper limit of normal value), renal dysfunction (serum creatinine > 141.4 µmol/L), recent history of infection, malignancy, previous history of peripheral vascular occlusive disease, autoimmune disease, or any other neurological disorders.

### Assessment of stroke severity and outcomes

All patients with AIS underwent brain magnetic resonance imaging (MRI) and angiographic studies (MR angiography, computerized tomography angiography, or conventional cerebral angiography). The severity of initial neurological deficits was assessed using the National Institutes of Health Stroke Scale (NIHSS). Functional neurological outcomes were assessed using the modified Rankin Scale (mRS) at discharge. Unfavorable outcomes were defined as mRS score > 2. Etiological stroke subtypes were determined according to the Trial of ORG 10,172 in Acute Stroke Treatment classification system [13].

### Data collection and laboratory examinations

All blood samples were obtained prior to treatment (with an anticoagulant and/or antiplatelet agent). Serum specimens were centrifuged, frozen within 2 h of collection, and stored at − 80 ºC until analyzed. The following concentrations of components were measured using the methods specified by the manufacturer: glucose, total cholesterol, HDL cholesterol, LDL cholesterol, TG, creatinine, uric acid, and HbA1c. Plasma sex hormone levels were also determined at the hospital laboratory using a biochemical analyzer (COBAS e 411 analyzer, Roche, Mannheim, Germany). E/T and E/free T ratios were defined as the ratio of estradiol to total testosterone and the ratio of estradiol to free testosterone, respectively.

### Statistical analyses

Statistical analyses were performed using SPSS (version 23.0; IBM SPSS Statistics, Armonk, NY, USA). Student’s *t-*test or the Mann-Whitney *U* test was used to compare continuous variables between the patient and control groups. The Chi-square or Fisher’s exact test was used to compare categorical variables. Values were expressed as mean ± standard deviation or number (percentage), as appropriate. Bivariate correlations between sex hormones and other parameters were compared using Pearson’s correlation test. Logistic regression analysis was used to determine the relationship between risk factors and AIS. The relation of risk factors with unfavorable functional outcomes (assessed by “at discharge” mRS score) was also investigated with the use of logistic regression models. After univariate analyses, odds ratios (ORs) with 95 % confidence intervals (CIs) were calculated using multivariate logistic regression. The goodness of fit of this model was assessed using the Hosmer and Lemeshow chi-squared statistic. All statistical tests were two-sided, and *P* < 0.05 was considered statistically significant.

## Results

Clinical and biochemical baseline demographic characteristics of the study participants are summarized in Table 1. A total of 146 patients with AIS were matched to 152 participants without the condition. The levels of SBP, DBP, total cholesterol, and TG were significantly higher, while the eGFR was significantly lower in the AIS group than in the control group. There were no differences between the AIS and control groups in BMI, DM, smoking, alcohol consumption, hyperlipidemia, HDL cholesterol, LDL cholesterol, and HbA1c. There were significant differences between the two groups in the percentage of HTN, cardiovascular disease, atrial fibrillation, and CKD. In addition, the characteristics of sex hormones are also presented in Table 1. There were no significant differences in the levels of estradiol, total testosterone, free testosterone, progesterone, and 17-OH progesterone between the groups (Table 1). However, the two groups differed significantly in the E/T ratio.

**Table 1:** Distribution of demographic and clinical characteristics among acute ischemic stroke patients and controls

	AIS (*n*=146)	Control (*n*=152)	*P*-value
Age, years	70.62 ± 10.67	70.51 ± 10.49	0.929
Systolic blood pressure, mmHg	147.12 ± 24.20	134.16 ± 22.60	<0.001
Diastolic blood pressure, mmHg	83.40 ± 19.18	75.32 ± 14.10	<0.001
Height, m	1.67 ± 0.07	1.64 ± 0.09	0.001
Weight, kg	65.80 ± 8.98	64.21 ± 10.72	0.167
Body mass index, kg/m^2^	23.55 ± 2.88	23.84 ± 3.27	0.413
Hypertension	77 (52.7)	58 (38.2)	0.014
Diabetes mellitus	39 (26.7)	28 (18.4)	0.097
Smoking, current	46 (31.5)	34 (22.4)	0.089
Alcohol, current	57 (39.0)	51 (33.6)	0.337
Cardiovascular disease^a^	32 (21.9)	19 (12.5)	0.033
Hyperlipidemia	61 (41.8)	49 (32.2)	0.094
Atrial fibrillation	26 (17.8)	14 (9.2)	0.040
CKD	33 (22.6)	17 (11.2)	0.013
Total cholesterol, mmol/L	5.24 ± 1.10	4.93 ± 1.06	0.014
Triglyceride, mmol/L	1.86 ± 0.99	1.60 ± 1.04	0.032
HDL-cholesterol, mmol/L	1.17 ± 0.26	1.23 ± 0.33	0.083
LDL-cholesterol, mmol/L	3.35 ± 1.04	3.15 ± 0.86	0.072
HbA1c, %	6.13 ± 1.64	5.83 ± 1.17	0.074
eGFR, mL/min/1.73 m^2^	69.93 ± 15.39	74.69 ± 13.64	0.005
Estradiol, pmol/L	71.63 ± 50.45	62.33 ± 49.70	0.110
Total testosterone, nmol/L	13.62 ± 7.66	15.53 ± 10.18	0.070
Free testosterone, nmol/L	0.25 ± 0.12	0.25 ± 0.09	0.827
Progesterone, nmol/L	1.71 ± 0.70	1.84 ± 0.63	0.098
17-OH progesterone, nmol/L	4.91 ± 1.84	4.96 ± 2.09	0.809
E/T ratio	6.75 ± 5.97	4.72 ± 4.38	0.001
E/free T ratio	366.43 ± 342.95	291.50 ± 332.19	0.056

Data are expressed as the mean ± SD, number (%)

*AIS* acute ischemic stroke, *CKD* chronic kidney disease, *HDL* high-density lipoprotein, *LDL* low-density lipoprotein, *eGFR* estimated glomerular filtration rate, *E/T ratio* estradiol-to-testosterone ratio, *E/free T ratio* estradiol-to-free testosterone ratio

^a^Cardiovascular disease includes coronary heart disease, heart failure, or peripheral arterial disease

All participants were categorized into tertiles according to the E/T ratio. When the baseline demographic and clinical characteristics of the participants were examined, there were significant differences between the tertiles in age, prevalence of AIS, SBP, BMI, DM, and current smoking and alcohol drinking habits (Table 2). Participants with the highest tertile E/T ratio were older and had higher SBP.

**Table 2:** Participant characteristics according to the estradiol-to-testosterone ratio tertile

	Tertiles of estradiol-to-testosterone ratio			*P*-value
	1st (<2.33)	2nd (2.37-6.39)	3rd (6.53<)	
	*n* = 99	*n* = 100	*n* = 99	
Age, years	69.42 ± 11.05	68.15 ± 10.73	74.13 ± 8.91	<0.001
Prevalence of AIS	33 (33.3)	50 (50.0)	63 (63.6)	<0.001
Systolic blood pressure, mmHg	134.84 ± 23.38	142.56 ± 25.18	144.11 ± 23.33	0.015
Diastolic blood pressure, mmHg	76.24 ± 15.67	80.70 ± 18.85	80.88 ± 16.79	0.100
Body mass index, kg/m^2^	24.13 ± 3.12	23.00 ± 3.22	23.97 ± 2.80	0.019
Hypertension	49 (49.5)	44 (44.0)	42 (42.4)	0.586
Diabetes mellitus	13 (13.1)	32 (32.0)	22 (22.2)	0.006
Smoking, current	25 (25.3)	19 (19.0)	36 (36.4)	0.020
Alcohol, current	32 (32.3)	30 (30.0)	46 (46.5)	0.032
Cardiovascular disease^a^	13 (13.1)	24 (24.0)	14 (14.1)	0.090
Hyperlipidemia	33 (33.3)	41 (41.0)	36 (36.4)	0.553
Atrial fibrillation	15 (15.2)	8 (8.0)	17 (17.2)	0.143
CKD	14 (14.1)	21 (21.0)	15 (15.2)	0.388
Total cholesterol, mmol/L	4.94 ± 1.14	5.02 ± 1.07	5.29 ± 1.03	0.060
Triglyceride, mmol/L	1.70 ± 1.02	1.63 ± 0.95	1.85 ± 1.08	0.307
HDL-cholesterol, mmol/L	1.21 ± 0.30	1.15 ± 0.31	1.24 ± 0.29	0.107
LDL-cholesterol, mmol/L	3.12 ± 0.96	3.26 ± 0.92	3.36 ± 0.98	0.202
HbA1c, %	5.72 ± 1.00	6.11 ± 1.77	6.10 ± 1.38	0.089
eGFR, mL/min/1.73 m^2^	74.64 ± 14.27	70.44 ± 14.16	72.02 ± 15.45	0.125
E2 (estradiol), pmol/L	26.69 ± 19.96	61.70 ± 36.44	112.32 ± 46.40	<0.001
Total testosterone, nmol/L	16.57 ± 10.87	16.09 ± 8.93	11.11 ± 5.68	<0.001
Free testosterone, nmol/L	0.24 ± 0.08	0.26 ± 0.10	0.24 ± 0.14	0.498
Progesterone, nmol/L	1.61 ± 0.54	1.91 ± 0.74	1.81 ± 0.66	0.004
17-OH progesterone, nmol/L	4.60 ± 1.81	5.33 ± 1.98	4.87 ± 2.05	0.030

Data are expressed as the mean ± SD, number (%)

*AIS* acute ischemic stroke, *CKD* chronic kidney disease, *HDL* high-density lipoprotein, *LDL* low-density lipoprotein, *eGFR* estimated glomerular filtration rate

^a^Cardiovascular disease includes coronary heart disease, heart failure, or peripheral arterial disease

The E/T ratio was associated with AIS in both crude and multivariable analyses. After adjustment for other confounding factors, multivariate logistic regression analyses found that participants with the highest E/T ratio were more likely to have AIS (OR: 3.084; 95 % CI: 1.616–5.886; *P* < 0.001) compared with those in the first tertile (Table 3). The adjusted OR of the highest E/T ratio was more obvious than that of SBP (OR: 1.016; 95 % CI: 1.003–1.029; *P* = 0.013) and HTN (OR: 1.750; 95 % CI: 1.026–2.984; *P* = 0.040).

**Table 3:** Univariate and multivariate analyses of risk factors for acute cerebral infarction

	Univariate analysis			Multivariate analysis		
	OR	95% CI	*P*-value	OR	95% CI	*P*-value
Systolic blood pressure	1.024	1.014-1.035	<0.001	1.016	1.003-1.029	0.013
Diastolic blood pressure	1.031	1.015-1.046	<0.001	1.015	0.998-1.033	0.085
Body mass index	0.969	0.900-1.044	0.412			
Hypertension	1.809	1.140-2.868	0.012	1.750	1.026-2.984	0.040
Diabetes mellitus	1.614	0.931-2.798	0.088			
Smoking, current	1.596	0.952-2.678	0.076			
Alcohol, current	1.268	0.790-2.036	0.325			
Cardiovascular disease^a^	1.965	1.057-3.654	0.033	1.374	0.660-2.863	0.396
Hyperlipidemia	1.509	0.940-2.421	0.089			
Atrial fibrillation	2.136	1.067-4.276	0.032	1.998	0.923-4.326	0.079
CKD	2.319	1.227-4.382	0.010	1.709	0.648-4.511	0.279
Total cholesterol	1.305	1.053-1.618	0.015	1.144	0.883-1.480	0.308
Triglyceride	1.288	1.018-1.631	0.035	1.313	0.997-1.728	0.053
HDL-cholesterol	0.510	0.237-1.098	0.085			
LDL-cholesterol	1.248	0.980-1.589	0.072			
HbA1c	1.164	0.984-1.377	0.077			
eGFR	0.977	0.962-0.993	0.006	0.989	0.965-1.014	0.397
E2 (estradiol)	1.004	0.999-1.008	0.110			
Total testosterone	0.976	0.950-1.002	0.075			
Free testosterone	0.791	0.099-6.339	0.825			
Progesterone	0.746	0.526-1.057	0.099			
17-OH progesterone	0.986	0.878-1.107	0.808			
E/T ratio						
1st tertile	Reference			Reference		
2nd tertile	2.000	1.128-3.547	0.018	1.813	0.955-3.442	0.069
3rd tertile	3.500	1.950-6.283	<0.001	3.084	1.616-5.886	0.001

*OR* odds ratio, *CI* confidence interval, *CKD* chronic kidney disease, *HDL* high-density lipoprotein, *LDL* low-density lipoprotein, *eGFR* estimated glomerular filtration rate, *E/T ratio* estradiol-to-testosterone ratio

^a^Cardiovascular disease includes coronary heart disease, heart failure, or peripheral arterial disease

Subsequently, logistic regression analyses were conducted to evaluate the risk factors related to early unfavorable outcomes (Table 4). In univariate logistic regression analyses, the E/T ratio had a statistically significant association with early unfavorable functional outcomes (*P* = 0.001). With adjustments for confounding factors, significant variables for unfavorable outcomes were age, HTN, atrial fibrillation, “on admission” NIHSS score, stroke subtype, and estradiol level. After adjusting for all other significant outcome predictors, the E/T ratio remained an independent unfavorable outcome predictor with an adjusted OR of 1.167 (95 % CI: 1.053–1.294; *P* = 0.003). The other significant variables for early unfavorable functional outcomes were old age (OR: 1.061; 95 % CI: 1.006–1.119; *P* = 0.029), and “on admission” NIHSS score (OR: 1.539; 95 % CI: 1.267–1.871; *P* < 0.001) (Table 4).

**Table 4:** Univariate and multivariate analyses for early unfavorable outcomes

	Univariate analysis			Multivariate analysis		
	OR	95% CI	*P*-value	OR	95% CI	*P*-value
Age	1.087	1.047-1.128	<0.001	1.061	1.006-1.119	0.029
Systolic blood pressure	0.994	0.981-1.008	0.405			
Diastolic blood pressure	1.001	0.984-1.018	0.886			
Body mass index	1.018	0.909-1.141	0.752			
Hypertension	1.985	1.024-3.848	0.042	2.601	0.849-7.963	0.094
Diabetes mellitus	1.981	0.941-4.170	0.072			
Smoking, current	1.076	0.535-2.166	0.837			
Alcohol, current	1.328	0.681-2.587	0.405			
Cardiovascular disease^a^	1.646	0.747-3.627	0.217			
Hyperlipidemia	1.889	0.970-3.677	0.061			
Atrial fibrillation	2.556	1.054-6.194	0.038	1.657	0.435-6.319	0.460
CKD	1.773	0.809-3.883	0.153			
Total cholesterol	1.001	0.994-1.009	0.750			
Triglyceride	0.998	0.994-1.001	0.221			
HDL-cholesterol	0.975	0.944-1.008	0.134			
LDL-cholesterol	1.003	0.995-1.011	0.481			
HbA1c	1.034	0.848-1.261	0.742			
eGFR	0.984	0.963-1.006	0.148			
Admission NIHSS score	1.682	1.395-2.029	<0.001	1.539	1.267-1.871	<0.001
Stroke subtypes						
Large artery atherosclerosis	Reference					
Cardioembolism	2.177	0.905-5.233	0.082	2.112	0.571-7.813	0.263
Small vessel disease	0.260	0.087-0.773	0.015	0.206	0.034-1.237	0.084
Unknown/undetermined causes	0.390	0.145-1.047	0.062	0.590	0.143-2.427	0.464
E2 (estradiol)	1.011	1.004-1.018	0.002	1.003	0.993-1.014	0.507
Total testosterone	0.961	0.919-1.005	0.080			
Free testosterone	0.169	0.012-2.450	0.192			
Progesterone	1.616	0.988-2.642	0.056			
17-OH progesterone	0.979	0.820-1.170	0.815			
E/T ratio	1.134	1.052-1.221	0.001	1.167	1.053-1.294	0.003

*OR* odds ratio, *CI* confidence interval, *CKD* chronic kidney disease, *HDL* high-density lipoprotein, *LDL* low-density lipoprotein, *eGFR* estimated glomerular filtration rate, *NIHSS* National Institutes of Health Stroke Scale, *E/T ratio* estradiol-to-testosterone ratio

^a^Cardiovascular disease includes coronary heart disease, heart failure, or peripheral arterial disease

**Fig. 1:**
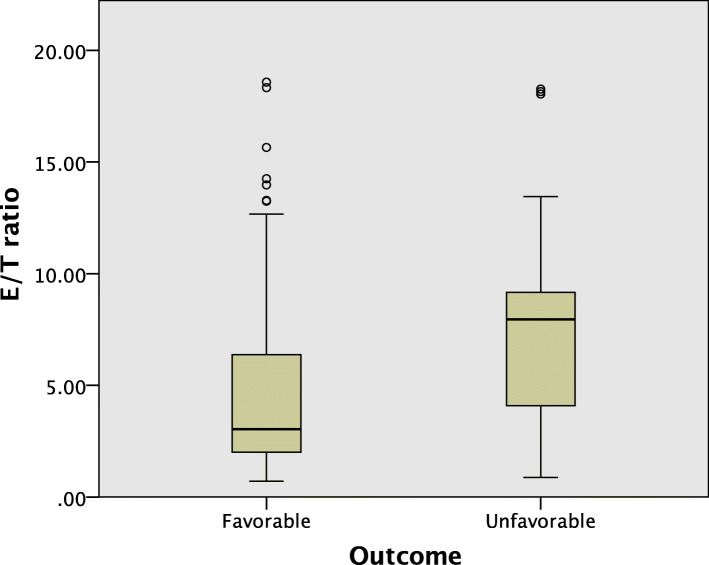
Boxplot of the estradiol/testosterone (E/T) ratio according to early functional neurological outcomes

The E/T ratio of the patients with early unfavorable functional outcomes was significantly higher compared to those with favorable outcomes (*P* < 0.001) (Fig. 1).

## Discussion

Our study demonstrated that the E/T ratio was independently associated with both AIS and poor early functional outcomes. In this study, the highest tertile of the E/T ratio was significantly associated with AIS. Our findings suggest that the E/T ratio may be a possible independent biological marker of AIS. Furthermore, an elevated E/T ratio may be used to predict early unfavorable functional outcomes. To the best of our knowledge, the current study is the first to investigate the inter-relationship between the ratio of sex hormones and early functional neurological outcomes in patients with AIS.

The presence of sexual dimorphism and their function has now been supported by evidence from the various fields of genetics, epigenetics, immunology, cellular physiology, and neurosciences. These mechanisms include apoptotic signaling cascades in neurons and glia, resident microglial activation, neuroglial response to ionic imbalance, autophagy, and mitochondrial toxicity [14]. In addition, the different effects of sexual hormone levels on the disease in males and females should be considered.

Clinical trials have demonstrated that estrogen increase the stroke incidence and severity in elderly women [2]. There is also evidence that estrogen therapy may increase thrombotic conditions and the risk of stroke. This suggests that hormones affect not only the brain, but also the coagulation system [15]. Similar trends were also observed in experimental stroke models. When administered to aged female rodents, estrogen paradoxically increased infarct volume after ischemic stroke [16]. These neurotoxic effects may be associated with a decrease in the utilization of insulin-like growth factor 1, a neuroprotective agent that decreases with age and is downregulated by estrogen treatment [17]. However, these studies were conducted only in women. Furthermore, the effects of estrogen therapy duration or dose on the experimental results were unclear [18].

Estradiol is the most potent endogenous estrogen. Estradiol is the main female sex hormone and plays an important role in the promotion of secondary sexual characteristics, and development of the reproductive system and breast tissue [10, 19]. Estradiol affects the brain through estrogen receptors (ER), most of which act through ERα of endothelium and smooth muscle cell, increase the transcription of pro-survival gene, and decrease pro-apoptotic gene [20–22]. Estradiol increases activation of extracellular signal-regulated kinase, which is involved in overall cell survival, inhibits the pro-apoptotic c-Jun N-terminal kinase pathway, and increases endothelial nitric oxide synthase and prostacyclin synthase, leading to vasodilation [20–23]. It is also known that estradiol may increase endothelial progenitor cell proliferation, which promotes angiogenesis [20]. Considering these mechanisms, estradiol is thought to have a neuroprotective effect basically, but some clinical studies have drawn different results from hypothesis. One study obtained blood samples from 302 post-menopausal women hospitalized with AIS and found an association between higher estradiol levels and stroke severity, 1-month mortality, and poor functional outcomes [24]. The study reported that estradiol levels remained independently associated with the aforementioned parameters, even after correction of confounders. The study concluded that stroke itself may elevate estradiol levels as an endogenous protective mechanism. Another study reported that higher circulating estradiol levels in late post-menopausal women were associated with higher mortality, independent of age and multiple confounders [25]. This study also suggested that the pro-thrombotic effects of estradiol may explain the high rate of thrombotic events observed in post-menopausal women. Lee et al. suggested that post-menopausal women with high endogenous free estradiol levels had 2.3-fold greater odds of stroke, independent of age [26]. However, these prospective studies can only reveal associations, not causality [24–26]. Nevertheless, higher levels of estradiol may be a marker of cerebrovascular disease and could help in identification of patients with AIS who are at risk of worsening unfavorable functional outcomes and mortality [24, 25].

Related studies of testosterone levels provide conflicting data on the true effects of the hormone on visceral adiposity, independent of other sex hormones. High serum testosterone is associated with reduced subcutaneous and increased abdominal adiposity in women, while low testosterone is associated with the same effect in men [27–29]. In older men, sex hormone levels do not decrease as rapidly as in menopausal women however, testosterone levels decline steadily with age [30].These declining testosterone levels have been reported to be associated with elevated visceral adiposity in older men [31]. This may play a role in increased risk of cardiovascular diseases in aging men. Low testosterone levels are associated with increased systemic inflammation [32] and endothelial dysfunction [33], both of which contribute to atherosclerosis. Epidemiological studies have shown that low testosterone levels independently predict the atherosclerosis development [34, 35]. Some meta-analyses have demonstrated that low testosterone levels increase the risk of major cardiovascular diseases as well as mortality [36, 37]. While many studies have reported the association between mortality in men and low circulating levels of testosterone [38], few have investigated the association between testosterone and the incidence of AIS. Levels of testosterone fluctuate throughout life in males. Increased levels observed in young men and decreased levels of the hormone in older men have been shown to increase the risk of ischemic stroke [39, 40]. It has also been hypothesized that aged men have reduced functional outcomes after AIS as a result of the progressive decreased testosterone, in contrast to the neuroprotective effect observed in the female brain [41]. Although the effects of estradiol and testosterone vary greatly depending on age and sex, it is reported that in elderly men, as total testosterone levels decrease, the incidence of stroke increases and functional outcomes may be poor even after adjusting for existing cardiovascular risk factors [42–44]. This is probably due to the anti-inflammatory and anti-oxidant effects of testosterone itself on brain endothelial cells and vascular smooth muscle cells in the pathologic state, but suggests that the balance between estradiol and testosterone may also be important [45, 46]. Based on these results, in order to determine the impact of testosterone on the outcomes of ischemic stroke, a more robust study should be designed that considers the levels of endogenous testosterone and other sex hormones.

We have suggested a unique concept in this study, namely the E/T ratio, and investigated the association between this ratio, AIS, and unfavorable functional outcomes. The novelty of this work is that it presents a new form of biomarker by combining estradiol and testosterone to quantitatively identify the influence of acute ischemic stroke. In this study, the E/T values showed clear differences between the two groups, but the values of estradiol and testosterone did not show statistical differences. This is probably considered a problem of diagnostic values. Although not statistically significant, there were an increase in estradiol and a decrease in testosterone level in AIS group, compared with control. The estradiol and testosterone levels were affected by age and sex. This study is also meaningful in comparing estradiol and testosterone respectively in patients with AIS, but more focused suggests that using the E/T ratio can increase the diagnostic value. This study suggests that estradiol and testosterone may have clinical significance in AIS patients, but lower testosterone level compared to estradiol level is more correlated with AIS and early unfavorable functional outcome. E/T ratio was concluded to be a useful biomarker for AIS and early unfavorable functional outcomes. The results of our study are consistent with some previously reported observations. In a study on middle-aged men, wherein testosterone or estradiol was measured by immunoassay, higher estradiol levels were associated with cardiovascular disease [47]. In a study on elderly men, higher total testosterone using mass spectrometry was associated with a lower incidence of cardiovascular events, with similar hazard ratio in men with and without pre-existing cardiovascular disease [48]. Another study in aged men using mass spectrometry for hormonal analysis found no association between testosterone and cardiovascular events, defined as myocardial infarction (MI), stroke, or cardiovascular disease-related death [49]. A recent study on elderly men reported that higher plasma testosterone is an associated biomarker for reduced risk of stroke, but not MI [44]. Another recent study reported that extremely low plasma testosterone levels were associated with a high risk of ischemic stroke in men, and that this was caused by being overweight, and HTN [50]. Testosterone was found to independently induce endothelial relaxation through secretion of endothelial nitric oxide synthase, which causes vasodilation [51]. In obese men, the reduction in these neuroprotective effects may be related to development of AIS, due to increase in blood pressure. Furthermore, the conversion of testosterone to estradiol arises in adipose tissue by aromatization, and excessive adipose tissue may reduce plasma testosterone concentrations [50, 52]. Therefore, the applicability of the E/T ratio, and the association between higher E/T ratio and prevalence of AIS should be considered.

Several limitations should be considered for the present study. First, this was a single-center study conducted on a single race. The sample size was small for the results may not be generalizable to other demographic groups. A large-scale, multi-center external validation of the current study is necessary. Second, we primarily considered cross-sectional observational data, which may reduce accuracy. Particularly, female were excluded from this study because plasma sex hormones vary widely depending on menstruation or menopause. Such variations are also observed in men. However, not to the extent seen in women. It would be interesting to conduct a study that distinguishes the causal relationship between sex hormones and stroke in pre- and post-menopausal women. Furthermore, it would be also interesting to conduct a large-scale study comparing reproductive and elderly age for various sex hormones in stroke. Third, we did not serially measure plasma sex hormone levels, which prevented us from determining the cause and effect relationship between the hormones and AIS or other functional outcomes. We did not illustrate the longitudinal relationship between sex hormones and AIS. Fourth, the E/T ratio may be related to inflammation and short-term outcomes, which may be related to the infarct volume, but could not be measured in this study. MRI volumetry should be used to provide more accurate and reliable information on infarct volume. A large-scale study using this technique is warranted. Fifth, access to the proven gold standard mass spectrometry estradiol measurement technique for routine clinical use is limited. Therefore, immunoassay, as used in this study, may have resulted in inaccurate measurement of low serum concentrations of estradiol in men, which could lead to overestimation of estradiol [53]. Sixth, there was no difference in BMI between stroke and control group in this study. In univariate analysis, BMI did not show any association with AIS or early outcome, and correlation analysis also showed no correlation with estradiol or testosterone. However, in several previous studies, BMI is known to be closely related to hormone level and particularly to estradiol level in postmenopausal women. BMI has been reported to be positively correlated with estrone concentrations in older estradiol therapy users [54], and there are studies showing that serum estrogen in post-menopausal women is directly associated with BMI [55, 56]. It is also known that elevated BMI alters the hypothalamus-pituitary-gonadal axis, hormone levels, and gametogenesis [57]. Therefore, a large scale studies including post-menopausal women are needed, considering the relationship between BMI and sex hormones. Finally, since early functional outcomes were assessed in this study, it is necessary to verify the association with long-term neurological outcomes. Despite these limitations, the results suggest that the E/T ratio may be of diagnostic and prognostic value for AIS.

## Conclusions

Our study demonstrated a relationship between the E/T ratio and AIS. The E/T ratio may be a useful, non-invasive, convenient indicator that potentially predicts early unfavorable functional outcomes in male patients with AIS. Further large-scale investigations are necessary to explore the causal associations among sex hormones, E/T ratio, AIS, and functional neurological outcomes. Estrogen and progesterone are neuroprotective and anti-inflammatory hormones in various disease models of the brain, particularly in acute inflammatory conditions such as stroke. Several recent studies have revealed conflicting data and indicate that androgens can either protect against or exacerbate ischemic damage. However, it remains to be determined whether sex hormones affect the acute stage of ischemic stroke. Thus, large scale additional studies are warranted to explore the potential mechanisms underlying this association and to separately evaluate reproductive age and elderly, including female patients.

## Data Availability

The datasets generated and/or analysed during the current study are available from the corresponding author on reasonable request.

## Abbreviations

AIS: Acute ischemic stroke
E/T: Estradiol/testosterone
E/free T: Estradiol/free testosterone
HTN: Hypertension
DM: Diabetes mellitus
CKD: Chronic kidney disease (CKD)
SBP: Systolic blood pressure
DBP: Diastolic blood pressure
HbA1c: Hemoglobin A1c
HDL: High-density lipoprotein
LDL: Low-density lipoprotein
TG: Triglycerides
eGFR: Estimated glomerular filtration rate
BMI: Body mass index
MRI: Magnetic resonance imaging
NIHSS: National Institutes of Health Stroke Scale
mRS: Modified Rankin Scale
ORs: Odds ratios
CIs: Confidence intervals
MI: Myocardial infarction
